# Production of dsRNA Sequences in the Host Plant Is Not Sufficient to Initiate Gene Silencing in the Colonizing Oomycete Pathogen *Phytophthora parasitica*

**DOI:** 10.1371/journal.pone.0028114

**Published:** 2011-11-23

**Authors:** Meixiang Zhang, Qinhu Wang, Ke Xu, Yuling Meng, Junli Quan, Weixing Shan

**Affiliations:** 1 College of Life Sciences, Northwest A&F University, Yangling, Shaanxi, People's Republic of China; 2 College of Plant Protection, Northwest A&F University, Yangling, Shaanxi, People's Republic of China; 3 State Key Laboratory of Crop Stress Biology for Arid Areas, Northwest A&F University, Yangling, Shaanxi, People's Republic of China; University of Aberdeen, United Kingdom

## Abstract

Species of the oomycete genus *Phytophthora* are destructive pathogens, causing extensive losses in agricultural crops and natural ecosystems. A potential disease control approach is the application of RNA silencing technology which has proven to be effective in improving plant resistance against a wide range of pests including parasitic plants, nematodes, insects and fungi. In this study, we tested the potential application of RNA silencing in improving plant disease resistance against oomycete pathogens. The endogenous *P. parasitica* gene *PnPMA1* and the reporter gene *GFP* were used to evaluate the potential application of host induced gene silencing (HIGS). The GFP-expressing *P. parasitica* efficiently colonized *Arabidopsis thaliana* lines stably expressing *GFP* dsRNA and showed no obvious decrease in GFP signal intensity. Quantitative RT-PCR analyses showed no significant reductions in the abundance of *GFP* and *PnPMA1* transcripts in *P. parasitica* during colonization of *A. thaliana* lines stably expressing *GFP* and *PnPMA1* dsRNAs, respectively. Neither *GFP* siRNAs nor *PnPMA1* siRNAs produced by transgenic plants were detected in *P. parasitica* re-isolated from infected tissues by Northern blot analyses. Phenotypic characterization of zoospores released from infected plant roots expressing *PnPMA1* dsRNA showed no motility changes compared with those from wild-type plants. Similar results were obtained by analysis of zoospores released from sporulating hyphae of *P. parasitica* re-isolated from *PnPMA1* dsRNA-expressing plant roots. Thus, the ectopic expression of dsRNA sequences in the host plant is not sufficient to initiate silencing of homologous genes in the colonizing oomycete pathogen, and this may be due to a number of different reasons including the absence of genetic machinery required for uptake of silencing signals in particular dsRNAs which are essential for environmental RNA silencing.

## Introduction

*Phytophthora* species belong to a group of eukaryotic microorganisms classified as oomycetes that are phylogenetically distant from true fungi [1]. Most *Phytophthora* species are destructive plant pathogens, and the most notorious species is *P. infestans* which was responsible for the Irish Potato Famine in the 1840s [2]. Due to their distinct physiological and biochemical characteristics, it is difficult to efficiently control the diseases caused by these pathogens [3]. Employment of resistance genes has been the most cost-efficient approach for disease control. However, loss of disease resistance in crop cultivars due to rapid virulence changes in pathogens, is widespread in many pathosystems, especially the diseases caused by oomycete pathogens. For example, all 11 major resistance genes identified so far in potato against *P. infestans* have been overcome by the pathogen [3]. Current disease control measures are largely dependent on application of chemicals, and novel approaches are urgently needed.

The infection process of host plants by *Phytophthora* pathogens include a typical biotrophic stage in which specialized structures called haustoria are developed [4], [5], [6]. Fungal haustoria function as physiological bridges through which host resources such as minerals, secondary metabolites and carbohydrates are translocated into the colonizing pathogenic fungi, but similar specializations for *Phytophthora* haustoria have not been demonstrated [7]. Translocation of macromolecules notably some effector proteins from pathogens into host plants via haustoria has also been shown as a possible mechanism responsible for induced susceptibility and disease development [8], [9].

The interaction between hosts and some parasitic plants also involves development of haustoria-like structures [10], but they are very different from those of fungi and oomycetes [4], [5], [6], [11]. Active transport of molecules through haustoria into the parasitic plant *Cuscuta pentagona* has been reported for a number of host plant-derived mRNAs, such as those encoding transcriptional factors and calmodulin proteins during the infection process [10], [12], [13], [14].

Double-stranded RNA (dsRNA) mediated RNA silencing is a conserved mechanism in many eukaryotes [15]. A key step of RNA silencing is the efficient processing of dsRNAs into short RNA duplexes of 21 to 28 nucleotides in length by a series of proteins associated with post-transcriptional gene silencing (PTGS), followed by the guided cleavage or translational repression of complementary mRNAs by the generated siRNA duplexes [15]. RNA silencing technology has become a useful experimental tool to study gene function in mammals [16], plants [17], nematodes [18], fungi [19] and oomycetes [20], [21].

It has been shown that transgenic expression of dsRNA in the host plant leads to silencing of target genes in the parasitic plant *Orobanche aegyptiaca*. *GUS* was used as a reporter to demonstrate that the translocation of silencing signals across haustoria was bi-directional, either from parasitic plant to the host or from host plant to the parasite [13]. Transgenic expression in the host plant tomato of a dsRNA construct homologous to the gene encoding mannose 6-phosphate reductase (M6PR) increased disease resistance. M6PR is a key enzyme in mannitol biosynthesis and important for the development of parasitic plant *O. aegyptiaca*. Further experiments showed that expression of endogenous *M6PR* mRNA in the parasitic *O. aegyptiaca* was down-regulated when interacting with the transgenic tomato [22]. These findings provided evidence that the silencing signals like dsRNAs and small interference RNAs (siRNAs) are translocated from host to parasitic plant cells and function in parasitic plants. It has also been shown that transgenic expression of dsRNAs in the host plants barley and tobacco has a silencing effect on homologous genes in the colonizing fungal pathogens *Blumeria graminis* [23] and *Fusarium verticillioides* [24], respectively, suggesting the potential of this novel strategy for control of fungal diseases.

Since the discovery of dsRNA-based gene silencing and its conserved mechanism in diverse eukaryotic organisms, significant progress has been achieved in demonstrating and applying gene silencing in *Phytophthora* [20], [25], [26], [27], [28]. Transgene-mediated internuclear gene silencing was demonstrated in *P. infestans* [25] transformants introduced with either sense or antisense gene constructs. Stable transgenic expression of dsRNA sequences has successfully silenced target genes in *P. infestans* [20], [26], [29] and *P. parasitica* [21]. Evidence for accumulation of siRNAs was also obtained in the silenced transformants of *P. infestans* [20]. The increased gene silencing efficiency in *P. infestans* by transgenic expression of dsRNAs [26] indicated that dsRNAs are critical triggers of gene silencing in *Phytophthora*. Furthermore, successful development [27] and application of transient gene silencing by exogenous introduction of dsRNAs into the *P. infestans* protoplasts [30], [31], [32] showed that *Phytophthora* could process exogenous dsRNAs and initiate a silencing effect. Gene silencing has been achieved in *P. parasitica* [21], [28], including the use of transgenic expression of dsRNAs [21].

In this study, we used *Arabidopsis thaliana-P. parasitica* as a model biotrophic pathosystem [5], [6] to examine the effect of transgenic expression of dsRNAs on the expression of homologous genes in the invading and colonizing oomycete pathogen. The green fluorescence protein (*GFP*) gene [33] was used as a reporter to examine changes in GFP signal intensities in the GFP-expressing *P. parasitica* during infection and colonization of *A. thaliana* engineered to express *GFP* dsRNA. The *PnPMA1* [34] gene from *P. parasitica* was used in this study as an endogenous target gene to monitor possible gene silencing in the pathogen induced by the host plant. The gene silencing effect in *P. parasitica* was evaluated by GFP fluorescence changes, followed by quantitative analyses of *GFP* transcripts at different infection stages in the host plant *A. thaliana*. Accumulation of gene-specific siRNAs generated and/or triggered by transgenic plants was further assessed in *P. parasitica* re-isolated from infected host tissues. Our results showed that ectopic expression of dsRNAs in the host plant *A. thaliana* was not sufficient to trigger RNA silencing of homologous genes in the colonizing biotrophic pathogen *P. parasitica*. This suggested that the oomycete pathogens may lack the genetic machinery required for uptake of silencing signals in particular dsRNAs during biotrophic interaction between the host plant and the pathogen.

## Results

### Small RNA-mediated gene silencing in *P. parasitica*

The endogenous *P. parasitica PnPMA1* (plasma membrane H^+^-ATPase1) gene was selected as an endogenous target in gene silencing experiments because it is highly expressed in zoospores and germinated cysts [35]. The gene encodes an atypical plasma membrane ATPase with an insertion of ∼155 residues in its C-terminus which is highly conserved in *Phytophthora* but not presents in the plasma membrane H^+^-ATPases of plants and true fungi [34].

**Figure 1:**
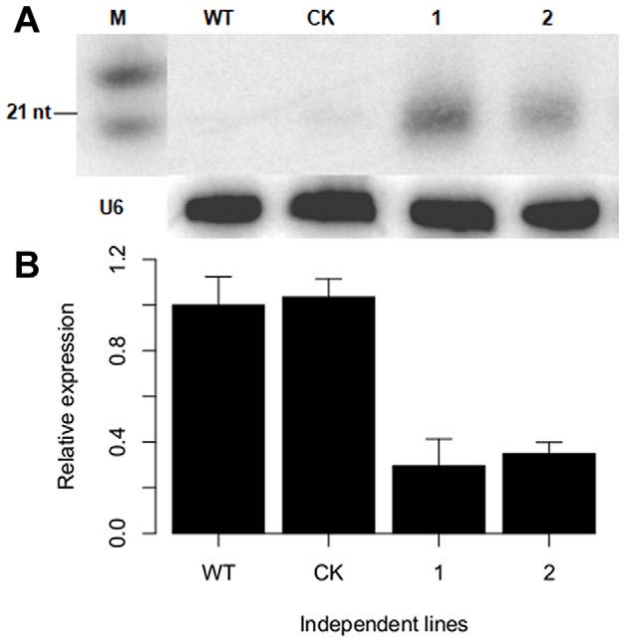
Analysis of *Phytophthora parasitica* transformants. (**A**) Detection of siRNA accumulation in transgenic *P. parasitica* lines transformed with a *PnPMA1* dsRNA construct. Northern blot of total RNA probed with *PnPMA1* specific probe. (**B**) Quantitative RT-PCR analyses of *PnPMA1* expression in *P. parasitica*. *P. parasitica WS041* was used as a reference gene for quantification and normalization of *P. parasitica* gene expression. The data presented are relative gene expression calculated after being normalized to *WS041* with the 2^−ΔΔct^ method. The data represent the means of three replicates. Bars represent the standard errors of three independent measurements. WT, wild-type *P. parasitica*; CK, *P. parasitica* transformed with hygromycin resistance gene; lanes 1 and 2, two independent *P. parasitica* silenced transformants accumulated *PnPMA1* siRNAs; M, RNA size marker, from bottom up are 21 and 24 nt RNAs, respectively.

Analysis of *P. parasitica* transformants that had been transformed with a dsRNA construct containing a partial *PnPMA1* sequence showed that 9 out of 60 transformants examined accumulated typical siRNA products of about 21 nucleotides (Figure 1A). This is consistent with the frequency of stable transformants generated by transformation of *P. parasitica* (WX Shan and AR Hardham, unpublished results). The silencing efficiency in these transformants ranged from 50% to 92% as determined by quantitative RT-PCR (data not shown). Heavy accumulation of *PnPMA1* siRNAs was detected in the most effectively silenced transformants. The relative *PnPMA1* expression levels in the two transformants showed in Figure 1B was 30% and 35%, respectively, of that the wild-type control. The result indicated that *P. parasitica* has functional gene silencing machinery for processing dsRNAs and production of gene-specific siRNAs, as shown in *P. infestans* [20]. This finding, together with the previously achieved transient gene silencing by exogenous introduction of dsRNAs into the protoplasts [27], [30], [31], [32] of *P. infestans* makes it reasonable to predict that exogenous dsRNAs such as those produced from the host plants once taken up by *P. parasitica* could be processed and initiate homologous gene silencing.

### Generation of transgenic *A. thaliana* expressing dsRNAs homologous to *GFP* and *PnPMA1*

To test the effect of dsRNAs produced in transgenic host plants on the expression of homologous genes in the colonizing *P. parasitica*, we chose *GFP* as a reporter gene which was widely used as a visual marker to test gene silencing efficiency [16]. The efficiency of gene silencing can be easily detected by measuring GFP fluorescence intensities [36], [37]. In addition, GFP has no toxic effect and has no homologous sequences in *A. thaliana*. These features make GFP superior to endogenous gene sequences which have potential off-target issues.

The endogenous *P. parasitica PnPMA1* was also selected as a target. Gene silencing experiments in *P. parasitica* showed that PnPMA1 protein is not essential for pathogen viability but is required for zoospore motility (MX Zhang and WX Shan, unpublished results). These features make *PnPMA1* a desirable target for examining potential effect of transgenic expression of dsRNAs in host plant on the pathogen development. In the case of successful uptake of silencing signals, the invading and colonizing *P. parasitica* may lose or display reduced zoospores motility.

**Figure 2:**
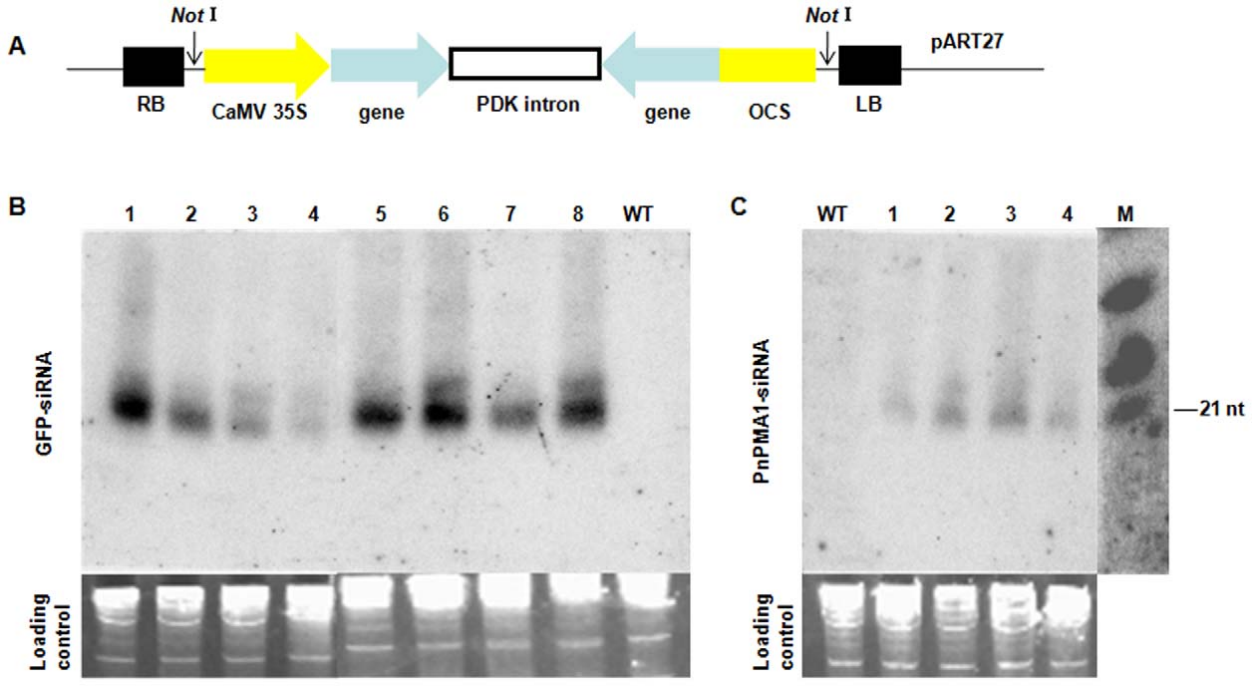
Generation of transgenic *Arabidopsis thaliana* lines with accumulation of siRNAs derived from transgene constructs. (**A**) Schematic representation of the silencing constructs in the pART27 binary vector. The selected regions of *GFP* (612 bp) and *PnPMA1* (262 bp) were cloned in an inverted-repeat configuration using the appropriate restriction enzymes into the sites spanned by 35 S promoter and OCS terminator regions in pKANNIBAL, respectively. The expression cassettes were subsequently transferred into the binary vector pART27, respectively. Accumulation of siRNAs in independent T2 *A. thaliana* lines transformed with (**B**) *GFP* dsRNA construct (lanes 1–8, T2 individuals from each of 8 independent T1 transgenic plants) (b1) or (**C**) *PnPMA1* dsRNA (lanes 1–4, T2 individuals from each of 4 independent T1 transgenic plants) construct; WT, wild-type *A. thaliana*; M, RNA size marker, 21, 24 and 30 nt from bottom up, respectively. Lower panel indicates ethidium bromide staining of total RNAs to show equal loading of RNA samples.

Transgenic *A. thaliana* plants expressing dsRNAs homologous to *GFP* and *PnPMA1* were successfully generated using a construct as shown in Figure 2A. Genomic PCR confirmed the presence of transgenes in the transgenic plants (data not shown). The successful expression of *GFP* and *PnPMA1* dsRNAs was confirmed by detection of siRNA accumulation in transgenic *A. thaliana* lines (Figure 2). Accumulation of *GFP* siRNAs was detected in eight transgenic lines but not in the non-transgenic line (Figure 2B). Similarly, *PnPMA1* siRNA accumulation was detected in four transgenic lines (Figure 2C). The size of the accumulated siRNAs was about 21 nucleotides, as indicated by the RNA markers. The accumulation of siRNAs varied among independent T2 *A. thaliana* transgenic lines and lines that accumulated more siRNAs were selected for further characterization. These transgenic *A. thaliana* lines appeared to be similar to that of non-transgenic plants in terms of their vegetative and reproductive growth as well as morphological characteristics.

### GFP signal intensity in the colonizing *P. parasitica* did not decrease in the *GFP* dsRNA-expressing transgenic *A. thaliana*

**Figure 3:**
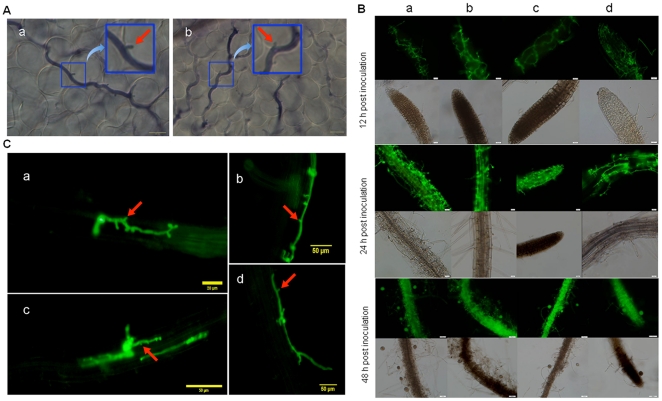
Cytological characterization of infection of transgenic *Arabidopsis thaliana* producing *GFP* dsRNA with GFP-expressing *Phytophthora parasitica*. (**A**) Successful colonization of both transgenic (a) and wild-type (b) *A. thaliana* plants by *P. parasitica* as shown with the development of haustoria in the mesophyll cells (scale bar: 20 µm). The diseased leaves of 3 d post inoculation were stained with trypan blue and viewed under Olympus BX-51 microscope equipped with differential interference contrast (DIC) optics. (B) Colonization of root tissues of transgenic *A. thaliana* lines expressing *GFP* dsRNA by *P. parasitica* stably expressing GFP (strain 1-1–2-1). The infected roots of both wild type (a) and three independent transgenic lines (b–d) that accumulated GFP siRNAs were collected 12 h, 24 h and 48 h post inoculation with zoospores prepared from *P. parasitica* strain 1-1–2-1, respectively, and were viewed with an Olympus BX-51 fluorescent microscope with a GFP filter (images taken 12 h and 24 h post inoculation, scale bar: 20 µm; images taken 48 h post inoculation, scale bar: 50 µm) (C) Development of *P. parasitica* haustoria in transgenic *A. thaliana* roots expressing GFP dsRNA. The infected roots of both wild-type (a) and three independent transgenic lines (b–d) that accumulated GFP siRNAs were collected 12 h post inoculation of zoospores prepared from *P. parasitica* strain 1-1–2-1 and were viewed with an Olympus BX-51 fluorescent microscope with a GFP filter (image of wild-type, scale bar: 20 µm; images of transgenic lines, scale bar: 50 µm).

To examine whether transgenic expression of *GFP* dsRNAs in *A. thaliana* affects its susceptibility to *P. parasitica* infection, detached leaves of *A. thaliana* were inoculated with zoospores of wild-type *P. parasitica* Pp016. Water-soaked lesions formed within 3 d post inoculation with no significant difference between transgenic and non-transgenic *A. thaliana* lines, and haustoria-like structures developed in both transgenic and wild-type *A. thaliana* leaves (Figure 3A). These indicated that transgenic expression of *GFP* dsRNA does not affect susceptibility of *A. thaliana* to *P. parasitica* infection.

Transgenic *P. parasitica* strain 1-1–2-1, which stably expresses ER-rendered GFP, was shown to be pathogenic on *Arabidopsis* (Figures 3B, 3C) and tobacco plants, the same as the wild-type strain Pp016 (WX Shan and AR Hardham, unpublished data). To determine whether transgenic expression of *GFP* dsRNA in *A. thaliana* affects GFP expression in the invading and colonizing *P. parasitica*, root inoculation assays were performed and infected tissues 12 h, 24 h and 48 h post inoculation were collected for microscopic characterization. The GFP fluorescence intensities in the colonizing hyphae of strain 1-1–2-1 showed no significant decrease throughout the infection stages compared with those infecting non-transgenic *Arabidopsis* lines (Figure 3B). We noticed that haustoria, which were considered to be the possible important structures where small RNA exchanges may occur, did not show GFP fluorescence decreases either (Figure 3C). We also noticed that multiple sporangia, which release zoospores and are important for the next cycle of infection, developed on the surface of *A. thaliana* roots 48 h post inoculation, and all the sporangia showed strong GFP fluorescent signals compared to that developed on the wild-type plants (Figure 3B, 48 h post inoculation). Zoospores released from these sporangia showed strong GFP fluorescence, indicating that GFP expression was not affected. These results suggested that the silencing signals in particular the dsRNAs produced by the host plant were not translocated across the cell membrane boundaries into the cytoplasm of the invading *P. parasitica*.

### The abundance of *GFP* and *PnPMA1* transcripts in *P. parasitica* were not affected during colonization in the transgenic *A. thaliana* lines

**Figure 4:**
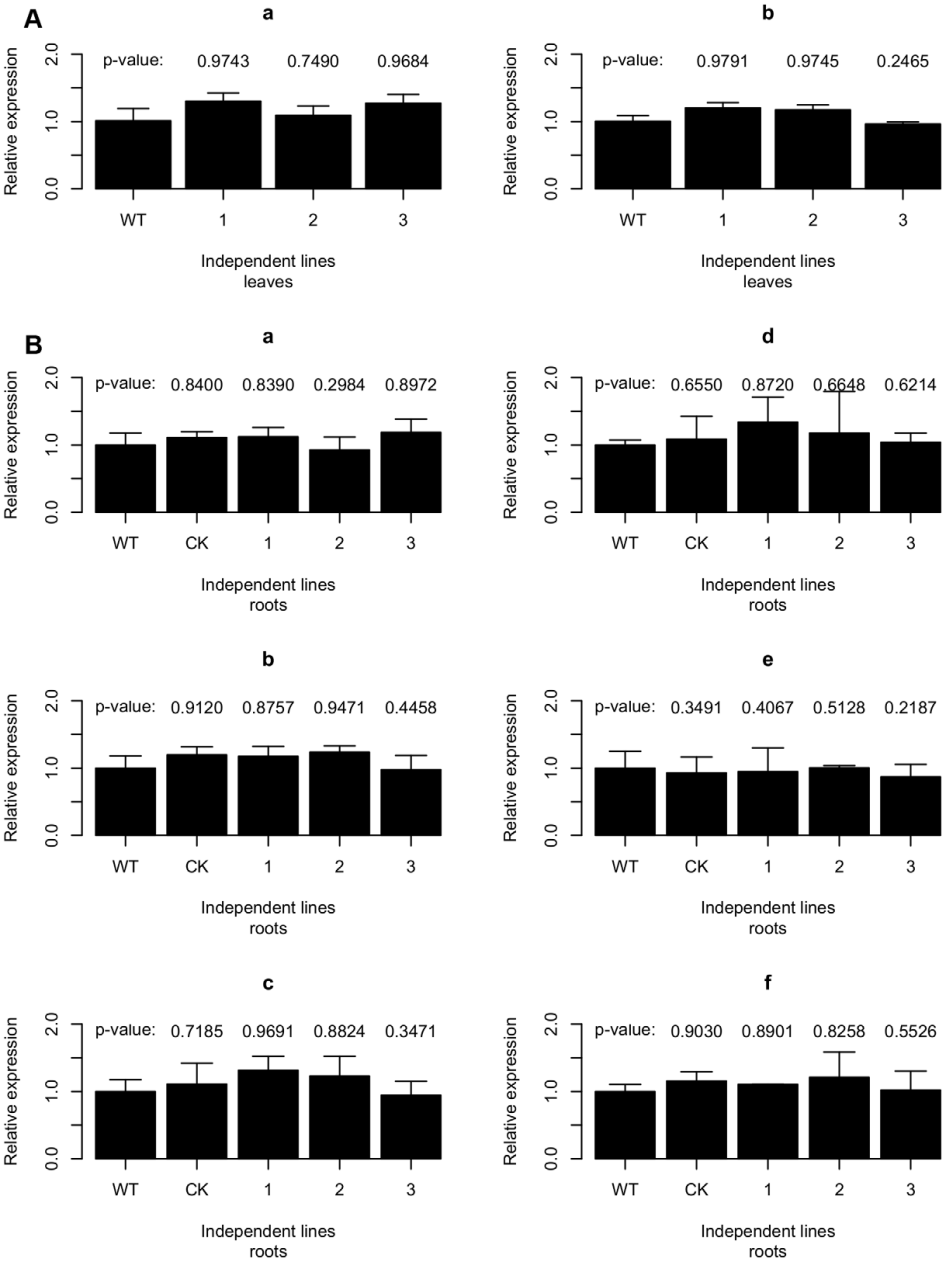
Quantitative RT-PCR analyses of *GFP* and *PnPMA1* expression in *Phytophthora parasitica*. (**A**) *GFP* (a) and *PnPMA1* (b) abundance in *P. parasitica* strain 1-2–1-1 stably expressing ER-rendered GFP in infected detached leaves. (**B**) Quantification of *GFP* (a-c) and *PnPMA1* (d–f) in 1-1–2-1 in infected roots 12 h, 24 h and 48 h post inoculation, respectively. WT, wild-type *A. thaliana*; CK, *A. thaliana* transformed with pART27; 1, 2 and 3 are three independent transgenic *A. thaliana* lines that accumulated *GFP* or *PnPMA1* siRNAs (the same set of lines as shown in Figure 2). *P. parasitica WS041* was used as a reference gene for quantification and normalization of *P. parasitica* gene expression. The data presented are relative gene expression calculated after being normalized to *WS041* with the 2^−ΔΔct^ method. The data represent the means of three replicates. Bars represent the standard errors of three independent measurements. Statistical significance of the target genes silencing effect was performed by Student's *t* test using *R* (one-tailed, transgenic lines versus wild-type).

Due to the potential stability of GFP [38], we performed quantitative RT-PCR analyses to further determine whether the silencing signals from the transgenic host plant were taken up by the colonizing *P. parasitica* and subsequently silenced the homologous genes in the pathogen. Transcription levels of *GFP* and *PnPMA1* in *P. parasitica* were evaluated both in infected detached leaves and roots. As for detached leaves, samples collected showed water-soaked lesions, a typical susceptible phenotype and were confirmed by trypan blue staining and cytological observation for compatible interaction. The quantitative RT-PCR assays showed that the abundance of *GFP* and *PnPMA1* transcripts was not significantly affected in the colonizing *P. parasitica* in transgenic *A. thaliana* leaves compared with that in non-transgenic lines (Figure 4A).

To further test the potential silencing effect on target genes, infected root tissues of three time points (12 h, 24 h and 48 h post inoculation with zoospores), which represent early, middle and late infection stages by microscopic observation, were used for gene expression analysis. The results showed that the expression levels of *GFP* and *PnPMA1* in *P. parasitica* were not affected, at all three stages tested (Figure 4B). We noticed that multiple sporangia developed on the surface of infected root tissues 48 h post inoculation, and the expression levels of target genes were also not affected at this stage. These results indicated that transgenic expression of dsRNAs in the host plant did not affect the expression levels of homologous genes in *P. parasitica* during plant infection.

### *PnPMA1*_siRNAs and *GFP*_siRNAs were not detectable in *P. parasitica* re-isolated from infected roots of transgenic *A. thaliana*

**Figure 5:**
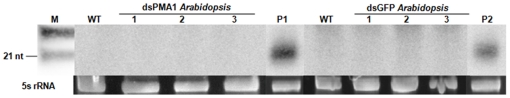
Detection of siRNA in *Phytophthora parasitica* re-isolated from transgenic plants producing *GFP* siRNAs or *PnPMA1* siRNAs. dsPMA1 and dsGFP *Arabidopsis*, *P. parasitica* re-isolated from transgenic plants expressing *PnPMA1* dsRNA and *GFP* dsRNA, respectively; WT, *P. parasitica* isolated from diseased wild-type *A. thaliana*; lanes 1–3, *P. parasitica* cultures five days after being isolated from three independent transgenic *A. thaliana* lines producing *GFP* siRNAs or *PnPMA1* siRNAs; P1, A *P. parasitica* transformant accumulated *PnPMA1* siRNAs as a positive control for *PnPMA1* siRNA detection; P2, A transgenic *A. thaliana* line accumulated *GFP* siRNAs as a positive control for *GFP* siRNA detection; M, RNA size markers, from bottom up are 21 and 24 nt RNAs, respectively.

RNA-dependent RNA polymerase (RdRP), which amplifies silencing signals, was present in the *Phytophthora* genome [20]. However, it is possible that the silencing efficiency was not sufficient in a limited time period to trigger significant reduction in gene expression and subsequent phenotypic changes. A previous report revealed that the greatest level of silencing was observed in *P. infestans* from 12 to 15 days after introduction of exogenous dsRNAs (17 days for GFP), though silencing was observed 1–4 days after dsRNA exposure [27]. To test this possibility, we performed re-isolation of the inoculated *P. parasitica* from the diseased root tissues and analyzed the re-isolated five day cultures for the presence and accumulation of homologous siRNAs by Northern blot and monitored the cultures for up to 30 days for GFP signal intensity. The results showed that neither *GFP* siRNAs nor *PnPMA1* siRNAs were detected (Figure 5), suggesting that no small RNA molecules derived from the host plant were taken up by the colonizing *P. parasitica*. Monitoring the re-isolated *P. parasitica* cultures for up to 30 days showed that there was no obvious decrease in GFP signal intensities (data not shown).

### Motility of *P. parasitica* zoospores produced from infected transgenic *A. thaliana* was not affected

We used *PnPMA1* as an endogenous target gene because our silencing experiments showed that *PnPMA1* is required for zoospore motility but not sporangia production and zoospore release in *P. parasitica* (MX Zhang and WX Shan, unpublished data). This easy-to-follow phenotype makes *PnPMA1* an ideal target for detection of whether the gene silencing could be initiated in *Phytophthora* through acquisition of host derived silencing signals like dsRNAs during intimate biotrophic interaction with host plants.

The root infection assay uses live seedlings and the root tissues can be heavily colonized with delayed resistance responses [6] and large numbers of sporangia are produced in root tissues 48 h post inoculation of *P. parasitica* zoospores. It also allows easy visualization of reduced GFP signal intensities in *P. parasitica* during infection and colonization. Diseased root tissues harboring sporangia were cold treated to release zoospores, and about 50 sporangia were closely monitored by light microscopy for the release of zoospores. The results showed no significant changes in the motility of zoospores released from the infected *PnPMA1* dsRNA-expressing transgenic plants compared with that of wild-type plants (data not shown), indicating that the endogenous *PnPMA1* was not effectively silenced in the colonizing *P. parasitica*.

Transient gene silencing in *P. infestans* exhibited a delayed decrease in mRNA abundance from 12 to 15 days after introduction of exogenous dsRNA [27]. To test the possible delayed silencing in *P. parasitica*, we re-isolated *P. parasitica* from diseased root tissues of the transgenic plants expressing *PnPMA1* dsRNA and monitored the re-isolated cultures for up to 30 days for their sporangia production and zoospore motility. No significant decrease in sporangia production and zoospore motility was observed (data not shown) indicating that, similar to that of *GFP*, the endogenous target gene *PnPMA1* was not effectively silenced in the colonizing *P. parasitica* in transgenic *A. thaliana*.

## Discussion

RNA silencing technology was reported to be effective in acquiring resistance against a wide range of pests including the fungal pathogens *B. graminis* [23] and *F. verticilliodes* [24]. In the present study, we tested whether this host-derived gene silencing approach is functional against *Phytophthora parasitica*, representative of a large group of pathogens called oomycetes. Our experimental data showed that transgenic expression of dsRNAs in the host plant *Arabidopsis* was not sufficient to trigger silencing of homologous genes in the invading and colonizing *P. parasitica*. The engineered expression of *GFP* dsRNA in host plants does not have a negative effect on the GFP fluorescence intensity in the invading *P. parasitica*, which is similar to that during colonization of the *Arabidopsis* wild-type. Furthermore, the transcription levels of target genes in the invading and colonizing *P. parasitica* were not affected in transgenic host plants producing homologous dsRNAs. This finding, together with the fact that *P. parasitica* has a gene silencing machinery and is able to process exogenously introduced dsRNAs, leads to fundamental questions that are associated with translocation of silencing signals in particular the dsRNAs from host plant into the *Phytophthora* pathogen.

It has been documented that ectopic expression of dsRNAs in plants led to silencing of homologous genes in parasitic plants, suggesting that there is successful translocation of silencing signals such as dsRNA or siRNA molecules from hosts to parasitic plants. Two possible models were proposed to explain the translocation through intimate specialized connection structures called haustoria between the host and parasitic plant: plasmodesmata or direct phloem connections [14]. Translocation of silencing signals is primarily achieved by cell-to-cell translocation via plasmodesmata followed by long distance transmission through the vascular system in the host plant. However, the RNAi strategy was shown to be less effective in maize against the parasitizing *Striga* [39]. Lack of strong evidence for the direct phloem connections via plasmodesmata between maize and *Striga asiatica* is probably responsible for the ineffectiveness of ectopic expression of dsRNAs in silencing the target genes in *S. asiatica*. This is probably the case for the plant-*Phytophthora* interaction in which there is no evidence to show the presence of plasmodesmata between the pathogen and host plant. To date, there has been no evidence to show the cross-kingdom plasmodesmata connections between the host plants and fungal or oomycete pathogens.

Gene silencing signals were shown to be capable of moving from host plants to parasitic nematodes and some insects and afterwards spreading systemically. In nematode *Caenorhabditis elegans*, *SID-1* and *SID-2* [40], [41], [42] were identified as involved in the uptake of dsRNAs and required for systemic RNAi. *SID* genes are widely present in nematodes, some insects [43], [44], and many animal genomes except that of the dipteran, plant [45] and oomycete *Phytophthora* genomes (data not shown). This suggests that *Phytophthora* may have no similar genetic machinery for uptake of dsRNAs which is essential for environmental RNA silencing.

In plants, systemic gene silencing is well-documented but knowledge of the identity of the mobile silencing signals is still limited [46]. Silencing signals such as siRNAs were processed from dsRNA substrates and their intercellular transmission was presumed to be through plasmodesmata [47]. However, whether the single-stranded RNA (ssRNA) or dsRNA mediates systemic silencing remains unclear [46]. Both single stranded siRNAs and siRNA duplexes were proposed to be mobile silencing signals [46], [48].

In the filamentous fungus *Aspergillus nidulans*, the target gene encoding ornithine decarboxylase was successfully silenced when the homologous double stranded siRNA was directly added to the liquid medium [49], indicating that *A. nidulans* is capable of taking up silencing signals from the environment. Genome analysis showed that there were no genes in *A. nidulans* homologous to the *SID* genes identified in *C. elegans* (data not shown), suggesting that *A. nidulans* may take up silencing signals such as siRNAs via alternative transmembrane delivery pathways. Similarly, expression of the reporter gene *GUS* in the transgenic necrotrophic fungal plant pathogen *F. verticillioides* was successfully down-regulated during infection of the tobacco host plant expressing homologous dsRNA [24]. More recently, transgenic expression of dsRNAs in the host plant led to silencing of homologous genes in the invading and colonizing *B. graminis* [23]. These results indicate that filamentous fungi, both necrotrophic and biotrophic pathogens, may share similar mechanisms for taking up dsRNAs or siRNAs from the environment.

Transient gene silencing experiments with protoplasts of *P. infestans* showed that exogenous supplement of artificial dsRNAs was sufficient to trigger silencing [27]. In this case, introduction of dsRNAs was assisted by the commonly used lipofectin agent. Our research showed that ectopic expression of dsRNA in the host plant was ineffective in triggering gene silencing in the colonizing *P. parasitica*, suggesting that translocation of *in planta* generated silencing signals such as dsRNAs from the host plant into the pathogen was ineffective. Internuclear systemic gene silencing in *P. infestans* was documented to be efficient [25], [50], largely because of the coenocytic nature of hyphae. This suggests that a clear, visible phenotype would be seen if the silencing signals were taken up by the colonizing hyphae and other infection-specific intimate structures such as haustoria where intensive exchanges of signals from both host plant and pathogen are present [7], [32], [51].

The recent report of successful silencing of genes in the invading and colonizing fungal pathogen *B. graminis* [23] by transgenic expression of homologous dsRNAs in the host plant suggested that the host plant was capable of exporting gene silencing signals, possibly including dsRNAs or fragments of them. Apart from phylogenetic distance, *Phytophthora* pathogens are also very different from pathogenic fungi in terms of interacting with host plants at the RNA-mediated signaling levels, and may lack the genetic machinery required for taking up silencing signals from the environment. Further experiments are needed to address the translocation of silencing signals from the host plant to the pathogen. For example, creation of transgenic *P. parasitica* expressing *C. elegans SID-1* or *SID-2* [40], [41], [42] which are involved in the uptake of dsRNAs and systemic RNAi, may be useful for understanding plant-oomycete interactions at the RNA level and for developing functional genomic tools to understand *Phytophthora* biology and pathology.

It is also possible that the silencing signals were successfully taken up by the pathogen but the silencing efficiency was not sufficient in a limited time period to trigger significant reduction in gene expression and subsequent phenotypic changes. In this research, we investigated the reduction of mRNA abundance using infection materials within 3 d post inoculation of *P. parasitica* zoospores. At this stage, water-soaked lesions were well developed and a significant proportion of inoculated leaves were colonized by the pathogen [6]. Experiments with *P. infestans* showed that for all three genes tested, the greatest level of silencing was observed from 12 to 15 days after introduction of exogenous dsRNAs (17 days for GFP), though silencing was observed 1–4 days after dsRNA exposure [27]. This was very different from other organisms in which gene silencing is typically activated within hours or 1–2 days and could persist for several days until normal gene expression is recovered [52], [53]. The delayed decrease in mRNA abundance is possibly a characteristic of gene silencing in oomycetes.

In conclusion, while the nature of silencing signal identities and their translocation are still not clear, our data indicates that ectopic expression of dsRNAs in host plants is not sufficient to trigger significant silencing of homologous genes in the invading and colonizing *Phytophthora* pathogens during an intimate biotrophic interaction with the host plant. Our results suggest that, unlike the successes of using silencing technology against fungal [23], [24] and nematode [54], [55] pathogens, the engineered resistance against *P. parasitica* pathogens through the production of homologous dsRNAs in the host plants would not be feasible.

## Materials and Methods

### *Phytophthora parasitica* strains and growth conditions

*Phytophthora parasitica* strain Pp016 (ATCC MYA-141) was originally isolated from diseased tobacco plants in Queensland, Australia. Strain 1-1–2-1 (WX Shan and AR Hardham, unpublished data) is a transformant of Pp016 stably expressing ER-rendered GFP [33]. All *P. parasitica* cultures were maintained on 5% (v/v) carrot juice agar (CA) supplemented with 0.002% (w/v) β-sitosterol and 0.01% (w/v) CaCO_3_. *P. parasitica* zoospores were prepared as described [6]. Routinely, 4×10^6^ zoospores were obtained from each 90-mm plate. Zoospore suspensions were filtered through one layer of Miracloth (Calbiochem, USA) and the concentration adjusted to 200 zoospores per µL for infection assays.

### Genetic transformation of *P. parasitica*

A *PnPMA1* hairpin structure with a stem length of a 262 bp was inserted into the *Sma* I sites of pTH210 vector [56]. The resultant construct was introduced into *P. parasitica* by co-transformation with pTH210 as described in Narayan, et al. [21]. To generate the GFP-expressing *P. parasitica*, the *GFP* gene amended with an ER-retention signal (HDEL) was inserted into the *Sma* I-linearized pTH210. The GFP expression cassette was amplified from the recombinant plasmid and introduced into *P. parasitica* by co-transformation with pTH210 [21].

### Plasmid constructs and plant transformation

The constructs used in this work are shown in Figure 2A. For preparation of the *GFP* dsRNA construct, a 612 bp fragment of GFP coding sequence [33] was amplified using primers dsGFP-F1 (CCG CTC GAG GCG ACG TGA ACG GCC ACA) and dsGFP-R1 (CGG GGT ACC CGA ACT CCA GCA GGA CCA T), and dsGFP-F2 (TGC TCT AGA GCG ACG TGA ACG GCC ACA) and dsGFP-R2 (ATA CCC AAG CTT CGA ACT CCA GCA GGA CCA T), and cloned into vector pKANNIBAL [57] in the sense direction at *Xho* I and *Kpn* I, and in the antisense direction at *Xba* I and *Cla* I, respectively. For preparation of the *PnPMA1* dsRNA construct, a 262 bp fragment of *PnPMA1* coding sequence was amplified by using primers dsPMA1-F1 (CCG CTC GAG ACG TGC ACC TCA ACT GGC TG) and dsPMA1-R1 (CGG GGT ACC ATC ATC TCA TTG TCG CGA GTC C), and dsPMA1-F2 (TGC TCT AGA ACG TGC ACC TCA ACT GGC TG) and dsPMA1-R2 (CCA TCG ATA TCA TCT CAT TGT CGC GAG TCC), and were inserted to pKANNIBAL in the sense direction at *Xho* I and *Kpn* I, and in the antisense direction at *Xba* I and *Cla* I, respectively. The expression cassettes constructed in pKANNIBAL [57], which include the dsRNA constructs for *GFP* or *PnPMA1* and the spanning CaMV 35S promoter and OCS terminator sequences, were released by *Not* I and cloned into the *Not* I site of binary vector pART27 [58], respectively. The resulting plasmids were introduced into *Agrobacterium tumefaciens* GV3101 by electroporation for plant transformation. *Arabidopsis thaliana* (ecotype Columbia-0, Col-0) transformation was achieved via the floral dip method [59]. Transgenic plants were initially identified by screening on 1/2 MS plates containing 50 µg mL^−1^ kanamycin, followed by further analyses by PCR to confirm introduction of transgenes.

### DNA extraction and PCR analyses

Genomic DNA from young leaves of 4-week-old T1 transgenic and non-transgenic *A. thalina* plants was extracted using the CTAB method [60]. An amount of 50 ng genomic DNA was used in the PCR reaction (20 µL). Gene specific primers were the same as that used in the silencing constructs. The PCR condition was as follows: an initial denaturation at 94°C for 5 min, followed by 30 cycles of 94°C for 30 s, 55°C for 30 s and 72°C for 40 s, with a final extension at 72°C for 5 min. PCR products were separated in 1% agarose gel and stained with ethium bromide.

### RNA extraction and siRNA detection

Total RNA was extracted from *Arabidopsis* or *P. parasitica* using TRIzol reagent (Invitrogen, USA) following manufacturer's protocol. A total of 15 µg total RNA was separated in a 15% polyacrylamide-7 M urea gel, and transferred onto Hybond N+ membranes (Amersham, UK) using a semidry blotter (BioRad, USA) at constant current (3 mA cm^−2^) for 20 min. After a brief rinse in RNase-free dH_2_O,the RNA was UV cross-linked to the filters at 1200×100 µJ cm^−2^ energy.

To detect siRNA in *Arabidopsis* or re-isolated *P. parasitica* (cultured in vitro for five days) by Northern, the filters were pre-hybridized for 3 h at 42°C in hybridization solution (0.2 M Na_2_HPO_4_, pH 7.2, 200 µg mL^−1^ denatured herring sperm DNA, 7% SDS). The whole 612 bp PCR fragment of *GFP* that was used for *GFP* dsRNA construct and the 262 bp fragment of *PnPMA1* selected for *PnPMA1* dsRNA construct were labeled using Random Primer DNA Labeling Kit (TaKaRa, China). The filters were hybridized overnight at 42°C. Membranes were washed twice for 15 min at room temperature with 2X SSC and 0.5% SDS before being exposed to the intensifying screens and scanned using a FLA-7000 Phosphimager (Fuji Photo Film Co., Ltd, Japan). Parallel loading of 21, 24 and 30 nt RNAs were used as size markers.

### Plant infection assays

Both detached leaf and whole seedling assays were employed and the inoculation methods were essentially as that described previously [6]. *A. thaliana* was grown at 20–25°C with a photoperiod of 12 h day/night. Detached leaves of seedlings of 28–30 days were placed on moist filter paper in a plastic tray and 20 µL of *P. parasitica* zoospore suspension (200 zoospores per µL) was drop-inoculated onto the abaxial surface. The inoculated leaves were kept in the dark at 25°C for three days before being harvested for microscopic characterization and RNA extraction.

For whole seedling root inoculation, *A. thaliana* seeds were sterilized and sown on 1/2 MS agar plates supplemented with 1% sucrose. The plates were kept in a growth chamber at 22°C under 12 h day/night photoperiod for two weeks. The seedlings were gently removed from the plates and inoculated by dipping roots into *P. parasitica* zoospore suspension for about 5 s, followed by transferring the seedlings onto 1/2 MS agar plates without sucrose. The inoculated seedlings were kept in a growth chamber for 12 h, 24 h and 48 h before being harvested for microscopic characterization as described above.

### Microscopic characterization of infection and colonization of transgenic plant by *P. parasitica*

The detached leaves of *A. thaliana* were collected 3 d post inoculation with *P. parasitica* zoospores and were stained with trypan blue. In brief, samples were transferred into 2 mL tubes each containing 1 mL of trypan blue solution (10 g of phenol, 10 mL of glycerol, 10 mL of lactic acid, 10 mg of trypan blue, dissolved in 10 mL of distilled water), followed by boiling the tubes in a water bath for approximately 3 min, and finally destained in 1 mL of chloral hydrate (2.5 g of chloral hydrate dissolved in 1 mL distilled water) overnight. The samples were mounted in distilled water and viewed under Olympus BX-51 microscope equipped with differential interference contrast (DIC) optics (Olympus, Japan).

For microscopic characterization of root infection, root tissues were collected 12 h, 24 h and 48 h post inoculation with zoospores of *P. parasitica* strain 1-1–2-1, which stably expresses ER-rendered GFP. The diseased roots were viewed under Olympus BX-51 fluorescent microscope with a GFP filter.

### Quantitative RT-PCR assay of gene expression in *P. parasitica* during plant infection

Total RNA was extracted using TRIzol reagent (Invitrogen, USA) from diseased leaf and root tissues separately as described above. Detached *A. thaliana* leaves were collected 3 d post inoculation of *P. parasitica* zoospores when clear water-soaked lesions developed, and infected root tissues were collected 12 h, 24 h and 48 h post inoculation, respectively. Total RNA was treated with DNase I (TaKaRa, China) before being used for cDNA synthesis. To generate first-strand cDNA, 0.5 µg DNase I treated total RNA was reverse transcribed in 10 µL volume using the PrimeScript^TM^ RT reagent kit according to the manufacturer's instructions (TaKaRa, China). Real-time quantitative PCR experiments were carried out using 5 µL of a 1∶25 dilution of the first-strand cDNA and SYBR Green, using SYBR Premix Ex Taq^TM^ II according to the manufacturer's instructions (TaKaRa, China). Expression levels of GFP and *PnPMA1* transcripts in *P. parasitica* were quantified using an iQ5 real-time cycler (BioRad, USA). Gene specific primer pairs (*WS041*: 5′-CAC GTA CAC ATG CCC GAG AC-3′ and 5′-TTC CCA TGT AGG CCG AGT ATT C-3′; GFP: 5′-CGT CCA GGA GCG CAC CAT CT-3′ and 5′-TGC GGT TCA CCA GGG TGT CG-3′; *PnPMA1*: 5′-ATG AGT GCC ACG ACT TCT TCC-3′ and 5′-GCA CGC TAC CCG TCA TCT C-3′) were designed and their specificities were validated by analyzing the melt curve, and *WS041* (GenBank accession number CF891677), a gene shown to be constitutively expressed throughout *P. parasitica* lifecycle stages was selected as a normalizing reference gene [35].
